# Clinical and radiological dissociation of anti-TNF plus methotrexate treatment in early rheumatoid arthritis in routine care: Results from the ABRAB study

**DOI:** 10.1186/1471-2474-15-251

**Published:** 2014-07-24

**Authors:** Péter Juhász, Ádám Mester, Anna-Julianna Biró, Gábor Héjj, Gyula Poór

**Affiliations:** 1 National Institute of Rheumatology and Physiotherapy, Budapest, Hungary; 2 School of PH.D. studies, Semmelweis University, Budapest, Hungary; 3 Clinic of Rheumatology, University of Medicine and Pharmacy, Târgu Mureş, Romania; 4 3rd Department of Internal Medicine, Chair of Rheumatology, Semmelweis University, Budapest, Hungary

**Keywords:** Rheumatoid arthritis, Anti-TNF plus MTX treatment versus MTX, Radiological progression, Clinical and radiological dissociation, Treatment outcome, Routine care

## Abstract

**Background:**

Rheumatoid arthritis (RA) is a chronic autoinflammatory joint disease which leads to the destruction of joints and disability of the patients. Anti-tumour necrosis factor (anti-TNF) drugs can halt radiological progression better than conventional DMARDs even in clinical non-responders.

**Methods:**

The efficacy of anti-TNF plus methotrexate (MTX) treatment versus MTX monotherapy on clinical and radiological outcomes were compared in early rheumatoid arthritis (RA) patients in clinical practice by retrospective analysis of an observational cohort.

49 early RA patients (group A) on first-line MTX monotherapy and 35 early RA patients (group B) on anti-TNF plus MTX treatment were selected from an observational cohort and evaluated retrospectively focusing on their first twelve months of treatment. Data on disease activity (DAS28) and functional status (HAQ-DI) were collected three monthly. One-yearly radiological progression was calculated according to the van der Heijde modified Sharp method (vdHS). Clinical non-responder patients in both groups were selectively investigated from a radiological point of view.

**Results:**

Disease activity was decreased and functional status was improved significantly in both groups. One-yearly radiological progression was significantly lower in group B than in group A. The percentage of patients showing radiological non-progression or rapid radiological progression demonstrated a significant advantage for group B patients. In addition non-responder patients in group B showed similar radiological results as responders, while a similar phenomenon was not observed in patients in group A.

**Conclusions:**

Clinical efficacy within our study was similar for tight-controlled MTX monotherapy as well as for combination treatment with anti-TNF and MTX. However MTX monotherapy was accompanied by more rapid radiological progression and less radiological non-progression. Anti-TNF plus MTX decreased radiological progression even in clinical non-responders supporting the advantage of anti-TNF plus MTX combination in dissociating clinical and radiological effects.

## Background

Rheumatoid arthritis (RA) is a chronic systemic inflammatory musculoskeletal disease that represents a significant health burden both with regard to comorbidities as well as to the mortality of patients [1,2]. The chronic inflammation and destruction of synovial joints leads to functional impairment, work loss and progressive disability [3]. In the past deeper insights into the pathogenesis of RA has led to the introduction of biologic agents and subsequently to considerable changes in the management of RA with respect to preventing and controlling disease progression [4]. The in-depth understanding of the disease course and the increasing data of treatment strategies supported the development of widely accepted recommendations on the management and therapy of the disease [5,6]. Clinical remission, prevention of joint destruction and long-term disability have emerged as the primary goals of modern treatment for RA [5].

The concept of the ‘window of opportunity’ supports that early agressive treatment can significantly change the long-term course of the disease, resulting in higher clinical response rates, less disability and less erosive damage [7,8]. Biological agents have proven to be effective in patients responding insufficiently to MTX in randomised controlled trials (RCTs), not only in reducing disease activity and improving functional status, but in slowing radiological progression [9-15]. Monoclonal antibodies against TNF including adalimumab, etanercept and infliximab, and lately the interleukin-6 receptor inhibitor tocilizumab and anti-CD20 rituximab prevented joint destruction even in patients failing to show a clinical response to MTX monotherapy [16-20]. Such “dissociation” between disease activity and radiological progression appears to be an additional advantage of biologics.

To the best of our knowledge as of yet the phenomenon of dissociation was evaluated only in RCTs, with selected patient populations and not in routine clinical care. Our aim was to examine how anti-TNF + MTX therapy affect clinical, functional and radiological outcomes – primarily focusing on dissociation - compared with MTX monotherapy in early RA patients in routine care.

## Methods

As part of the ABRAB (Assessment of Biologics in Rheumatoid Arthritis in Budapest) study, a retrospective analysis of an observational cohort, adult (≥18 years) early RA patients (diagnosed ≤2 years) from the observational cohort of the outpatient clinic of the National Institute of Rheumatology and Physiotherapy, Budapest was performed. All patients were diagnosed according to the 1987 American College of Rheumatology criteria [21]. Patients were selected randomly based on the availability of baseline and 12 month radiographs of hands and feet in order to calculate radiological progression after 12 month. High baseline disease activity (DAS28 ≥ 5.1) was also among the selection criteria. Approval of the Ethics Committee of National Institute of Rheumatology and Physiotherapy in Budapest was given prior to the study, and informed consent was obtained from all patients.

We grouped selected patients into two groups: patients in group A (n = 49) received first-line MTX monotherapy (10–20 mg weekly) with no previous DMARDs, while those in group B (n = 35) received anti-TNF + MTX (10–20 mg weekly) treatment. Patients in group B were treated with the following agents: infliximab (20%), etanercept (22,9%), adalimumab (40%), golimumab (14,2%) and certolizumab (2,9%). All anti-TNFs were administrated according to the method of administration indicated in the summary of product characteristics. All patients in group B failed one or two prior DMARDs according to local guidelines on the use of biologics in RA.

Patients in both groups were treated in routine care. All patients had erosive disease, as documented by having at least one erosion on baseline hands and feet X-rays.The baseline characteristics of the two treatment groups were comparable and shown in Table 1.

**Table 1:** Baseline demographic and disease characteristics*

	**Group A (n = 49) (MTX treatment) % v. mean (SD)**	**Group B (n = 35) (anti-TNF + MTX treatment) % v. mean (SD)**
Age, (mean ± S.D.)	60.1 (13.7)	54.7 (15)
Women, n (%)	42 (85.7%)	28 (80%)
Disease duration, (month)	7.9 (2)	8.5 (1.2)
IgM RF positive, n (%)	34 (69.4%)	22 (62.9%)
Anti-CCP positive, n (%)	37 (75.5%)	26 (74.3%)
DAS28ESR , mean ± S.D.	6.1 (0.9)	6.4 (0.9)
CDAI, mean ± S.D.	36.2 (11.5)	33.6 (12.7)
Tender joint count, mean ± S.D.	13.1 (6.5)	14 (7.2)
Swollen joint count, mean ± S.D.	7.5 (5.9)	9.4 (5.2)
Patient global assessment (VAS 0–100), mean ± S.D.	65 (17.9)	64.3 (18.3)
ESR, mean ± S.D.	43.7 (22.4)	46.9 (33.5)
CRP, mean ± S.D.	27.5 (28.4)	40.3 (46.9)
HAQ-DI (0–3), mean ± S.D.	1.4 (0.7)	1.6 (0.7)

*There were no statistically significant differences in any demographic or baseline characteristics between the groups, by Mann–Whitney test for continuous variables and chi-square test for dichotomous variables; statistical significance was set as p < 0.05.

(RF: rheumatoid factor, anti-CCP: anti-cyclic citrullinated protein, DAS28ESR: disease activity score based on the erythrocyte sedimentation rate, CDAI: clinical disease activity index, VAS: visual analogue scale, ESR: erythrocyte sedimentation rate, CRP: C-reactive protein, HAQ-DI: Health Assessment Questionnaire - Disability Index, vdHS: van der Heijde modified Sharp score).

Clinical data was recorded at five timepoints during the first 12 months of treatment in both groups (at 0, 3, 6, 9 and 12 month). Disease activity was measured by DAS28 based on the erythrocyte sedimentation rate and by CDAI (clinical disease activity index), functional status was determined using HAQ-DI score. We evaluated the efficacy of anti-TNF + MTX treatment and MTX monotherapy in reducing disease activity and improving functional status in both groups in the first 12 month of their investigated treatment period, and also compared the differences between their data. The ratio of patients reaching the state of remission or low disease activity was also determined in both groups. Remission was defined as DAS28 < 2.6 or CDAI ≤2,8 and low disease activity was defined as DAS28 ≤ 3.2 and ≥2.6 or CDAI ≤10 and ≥2.9. [5].

All patients had hands and feet radiographs at baseline and at the end of the 12th month treatment period. Radiological progression was measured according to the van der Heijde modified Sharp method (vdHS; 0–448 points) on radiographs of the hands and feet. All radiographs were scored by three readers, a radiologist and two rheumatologists (AM, JB, PJ) blinded to patient identity, treatment and the date of the radiographs. Radiological values given by agreement of the three readers were used for the analysis. As a consequence of routine clinical care, the interval between the two different radiographic timepoints were for some patients less or more than 12 months (13 ± 2,4). In these cases radiological progression at 12 months (vdHS U/year) was calculated dividing the change in the radiological score with the ratio of the real time interval and the assumed 12 months. The impact on radiological progression was compared between the different treated groups, and in addition the ratio of patients with no radiological progression (vdHS = 0 U/year) and those showing rapid radiological progression (vdHS ≥ 5 U/year) was also compared between the two groups [22]. To investigate the dissociation between reduction of disease activity and impact on radiological progression in groups, radiological progression at 12 months as well as the ratio of radiological non-progression and that of rapid radiological progression was compared in patients who failed to show a clinical response. Clinical non-response was defined as a time-averaged DAS28 ≥ 3.2. The time-averaged DAS28 was calculated as the mean DAS28 of visits at month 3, 6, 9 and 12.

Statistical analysis was performed with Graphpad Prism 5.00 statistical software. The distribution of the variables is provided as mean ± SD. We used the independent two-tailed Student’s t-test and paired t-test to compare continuous variables between and in groups. We used either Mann–Whitney or Wilcoxon matched pairs test for non-parametric data, while one-way or repeated measures ANOVA with corresponding post-tests (Tukey’s or Dunnet’s multiple comparison) was used for comparing three or more variables. We used Fisher’s exact test to compare dichotomous variables between groups and considered p < 0.05 as statistically significant.

## Results

### Disease activity reduction

**Figure 1:**
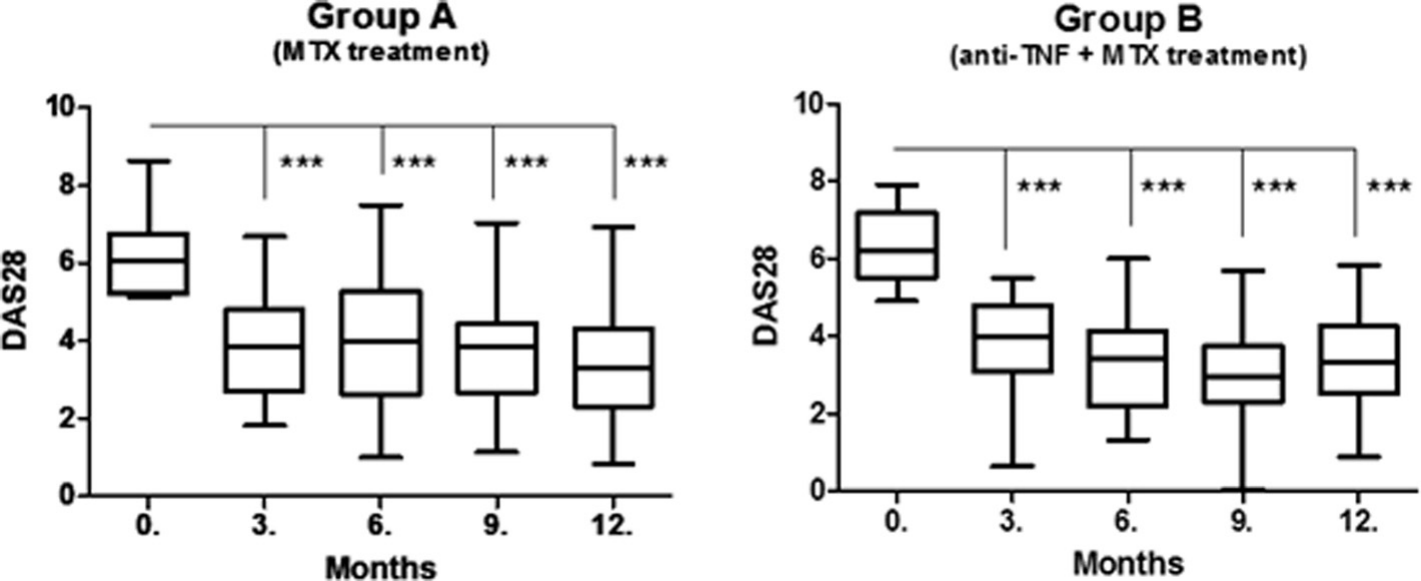
**Disease activity (DAS28) at 0, 3, 6, 9 and 12 months in group A and group B.** (mean, 25&75 percentiles and min.- max. values) ***: p < 0,001.

Mean disease activity (DAS28) was significantly reduced compared to the baseline mean value at all visits both in group A and group B (p < 0.001). From the month 3 visit onward, no further significant improvement was observed in either group (Figure 1). Mean disease activity reduction was greater in number at all visits for group B, but significant differences were found only between the 0–6 and 0–9 month timepoints when comparing both groups.

### Reaching DAS28 remission and the state of low disease activity

There was no significant difference in the percentage of patients reaching DAS28 or CDAI remission or low disease activity between the two groups at any of the visits (data not shown). At 12 months 15 (30.6%) and 11 (31.4%) patients showed DAS28 remission, 9 (18,37%) and 8 (22,86%) patients showed CDAI remission in group A and B respectively, with an additional 7 (14.3%) and 5 (14.3%) patient achieving DAS28 low disease activity, 19 (38,77%) and 17 (48,57%) patients achieving CDAI low disease activity respectively.

### Functional status improvement

The mean functional status (HAQ-DI) was significantly reduced compared to the baseline mean value at all visits both in group A and group B (p < 0.001). Similarly to disease activity, we could not observe a significant improvement after 3 months (data not shown).

### Overall radiological results

**Figure 2:**
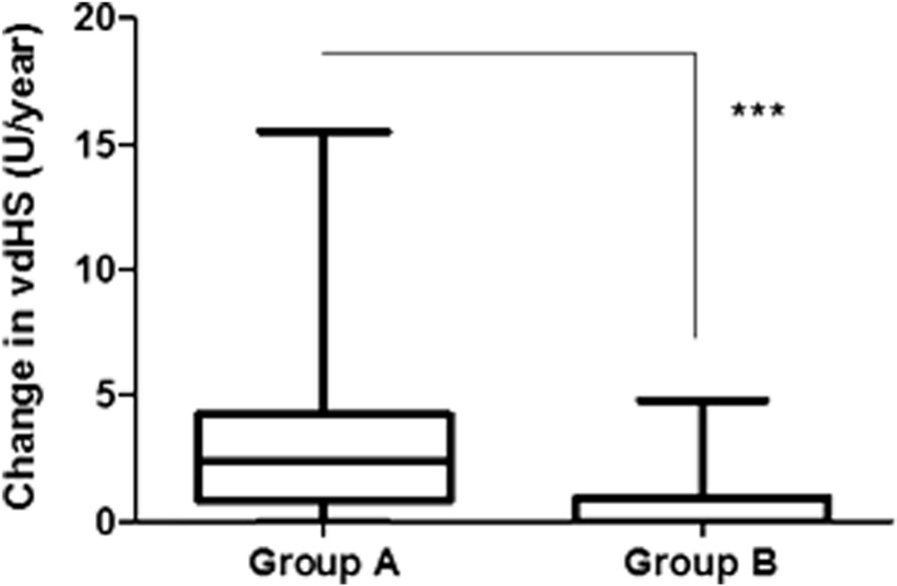
**Radiological progression (vdHS U/year) at 12 months in group A and group B.** (mean, 25&75 percentiles and min.- max. values) ***: p < 0,001.

Using the vdHS scoring method the mean yearly radiological progression was 3.177 (3.453) U/year in group A (95% CI 2.185-4.169) and 0.7071 (1.117) U/year in group B (95% CI 0.3234-1.091) with statistically significant difference (p < 0.001) as shown in Figure 2. The percentage of patients showing rapid radiological progression (vdHS/year ≥ 5 U/year) or radiological non-progression (vdHS/year = 0 U/year) is shown in Table 2. None of the patients in group B showed rapid radiological progression. Radiological progression was halted in a high percentage (n = 19, 54,3%) of these patients, which was statistically significantly different to as compared to patients in group A (p < 0.001).

**Table 2:** Number and percentage of patients showing radiological non-progression or rapid radiological progression in group A and group B

	**Group A (n = 49)**	**Group B (n = 35)**	**p-value**
Radiological non-progression n(%)	9 (18.4%)	19 (54.3%)	***
Rapid radiological progression n (%)	9 (18.4%)	0 (0%)	**

***: p < 0.001; **: p < 0.01.

### Radiological progression in clinical responders and non-responders

**Figure 3:**
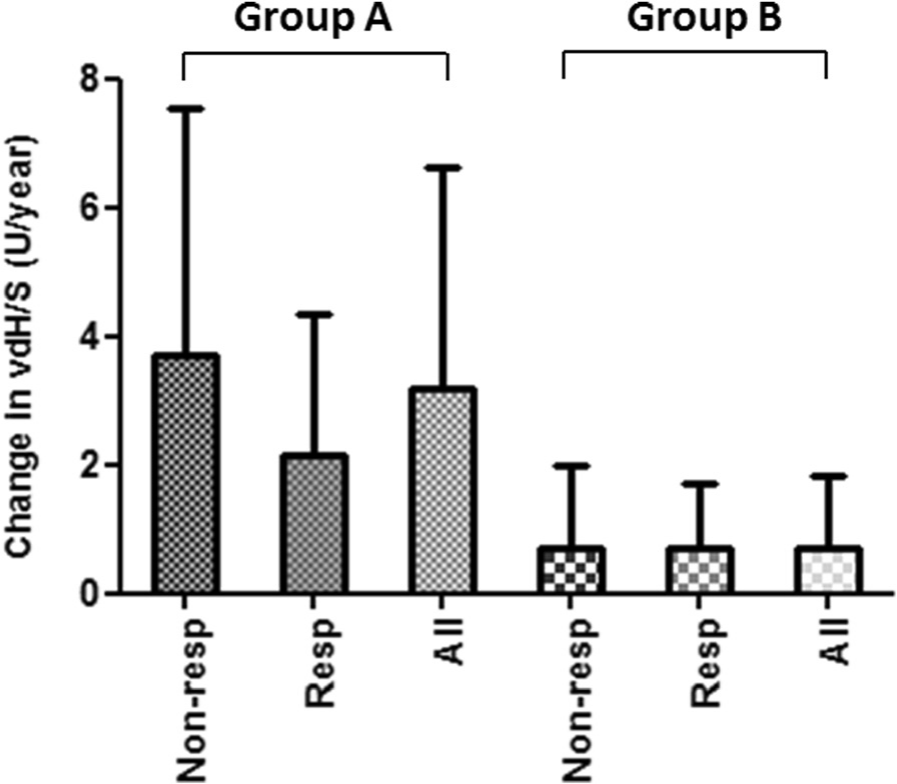
**Change in radiological progression (vdHS U/year) in group A and group B regarding all, clinical non-responder and clinical responder patients.** (All: all patients, Resp: clinical responder patients, Non-resp: clinical non-responder patients).

**Figure 4:**
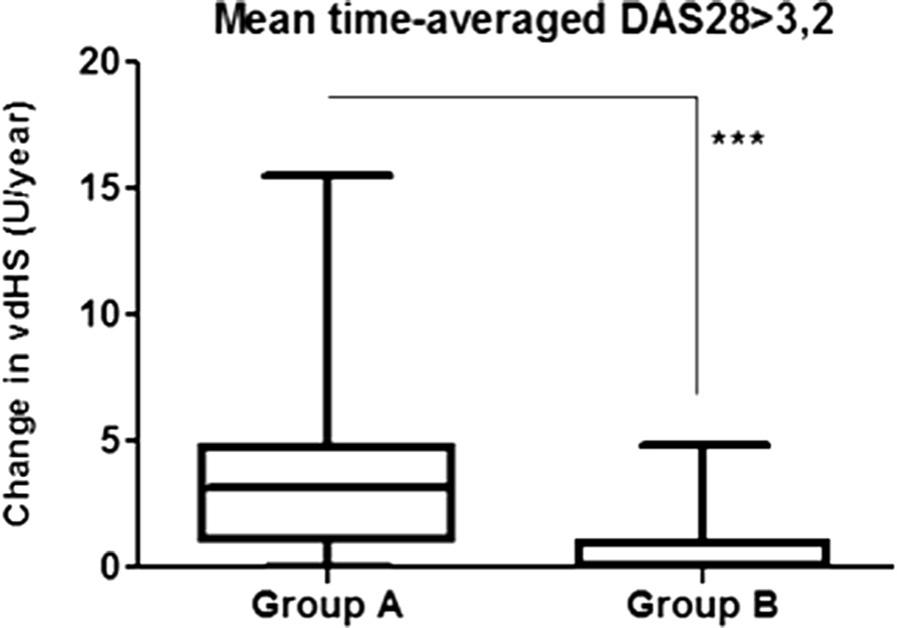
**Radiological progression at 12 months (vdHS U/year) of clinical non-responders (time-averaged DAS28 > 3.2) in group A and group B.** (mean, 25&75 percentiles and min.- max. values) ***: p < 0.001.

To perform a more detailed evaluation of radiological progression at 12 months patients in both treated groups were divided into subgroups of clinical non-responder and clinical responder patients. A non-significant trend was observable in group A showing that radiological progression was higher in clinical non-responders than in clinical responders when compared to all patients within that group. This suggests the persistant effect of disease activity on radiological progression on MTX monotherapy. In group B we observed no difference in radiological progression at 12 months between responders and non-responders, indicating that anti-TNF + MTX treatment indeed dissociates disease activity and radiological progression (Figure 3).Radiological progression at 12 months and the percentage of patients showing radiological non-progression or rapid radiological progression were separately assessed in the subgroups of clinically non-responder patients. Within these subgroups mean radiological progression was 3.698 (3.837) U/year (95% CI: 2.337-5.058) in group A and 0.7141 (1.26) U/year (95% CI: 0.066-1.362) in group B (p < 0.001, Figure 4). Group B was associated with a higher percentage of patients with radiological non-progression than group A (58.8% vs 12.1% respectively, p < 0.001). We found no significant difference regarding rapid radiological progression, nevertheless group B showed better results than group A in this respect as well (0% vs 21.2% respectively, p = 0.07).

## Discussion

We evaluated early RA patients under different treatment in routine rheumatological outpatient care and showed comparable clinical efficacy of anti-TNF + MTX and MTX monotherapy in the first 12 month of treatment. Both groups had significant improvement in DAS28 reduction which was noticeable at the 3 months visit, and was sustained along the observed 12 months treatment period. The value of DAS28 reduction was higher at all visits in the anti-TNF + MTX treated group, and was significant at 3 months. The percentage of patients achieving DAS28 remission as the desirable therapeutic goal in RA [5], was comparable between the anti-TNF + MTX and MTX monotherapy treated groups, despite the higher DAS28 reduction in the former group. The rate of remission at month 12 was 31.4% in the anti-TNF + MTX treated group consistent with data found in the literature on patients treated in clinical practice [23,24]. Similar rates of DAS28 remission in the MTX-treated group (30.6%) can be explained by the early, tight controlled design of the treatment.

Functional status (HAQ-DI) was significantly improved in both groups from month 3 and was sustained along the observed 12-month treatment period, with no clear differences between the two groups. Functional disability, as measured by HAQ-DI has been shown to have two components. The irreversible component correlates with radiological status, mainly cartilage destruction and subluxation, and worsens with time. The reversible domain is related to disease activity and can be therapeutically influenced [25,26]. Our observed groups had comparable baseline radiological status as well as comparable improvement of clinical activity that explains the similar effect of anti-TNF + MTX and MTX monotherapy on functional status.

There is strong evidence on the efficacy of anti-TNF + MTX vs. MTX monotherapy in slowing/halting radiological progression [9-13]. Comparing the two groups, anti-TNF + MTX treated patients had significantly less radiological progression than those treated with MTX monotherapy. Our data confirms the superiority of anti-TNF + MTX treatment not only in decreasing radiological progression, but also in inhibiting rapid radiological progression and increasing the rate of radiological non-progression. The high percentage of radiological non-progression in the anti-TNF + MTX treated group indicates marked clinical success since the investigated patients had baseline radiographic damage and anti-CCP positivity in high percentage that are the main predictive factors of poor radiologic outcome [27].

Erosions of the joints and cartilage destruction are hallmarks of RA. Formerly, it was conceptualized that in RA inflammation leads directly to the destruction of bone and cartilage. Recently we have learned that bone destruction is intricately linked to the RANKL (receptor activator of nuclear factor kB ligand)-OPG (osteoprotegerin) system which is strongly, but not exclusively connected to the inflammatory processes occurring within the joint [28]. The disbalance between RANKL and OPG mainly determines the degree of proliferation and activity of osteoclasts in RA. Osteoblasts produce both RANKL and OPG, the ratio of which is influenced by hormones, growth factors and many cytokines, among them TNF-alpha [28]. The strong inhibitory effect of anti-TNFs on radiological progression may be explained by their direct inhibitory effects on osteoclast activity, in addition to their overall anti-inflammatory effect [16].

RCTs (randomized controlled trials) comparing anti-TNFs + MTX to MTX support the phenomenon of dissociation between inflammation and radiological progression, which may explain the slowing/halting of radiological progression in the absence of a clinical response [16-18]. Dissociation may potentially be explained by the threshold hypothesis, which assumes that TNF-driven osteoclast activation occurs only when the level of TNF reaches a putative threshold. In case of clinical and radiological dissociation anti-TNF therapy decreases the level of TNF below this threshold, but not low enough to cease the inflammatory activity as well [29]. This assumes that inflammation is sustained at lower levels of TNF than at levels at which osteoclast activation occurs. Recent analysis from the PREMIER trial showed that the dissociation between clinical and radiological outcomes (joint space narrowing and joint erosions) at week 52 and 104 occurred in patients treated with adalimumab (ADA) + MTX but only to a mild degree in patients on ADA monotherapy [30], which might indicate that MTX may enchance the dissociative potential of ADA treatment. Dissociation has also been shown for IL-6 inhibition with tocilizumab [19] and with anti-CD20 rituximab therapy [20], A recent meta-analysis of intensive DMARD combination therapy with step-down prednisolone study also suggested a similar phenomenon in patients [31].

Nevertheless, there are some limitations to our clinical study. Firstly the anti-TNF + MTX treated group received preliminary DMARDs that decreases comparability with the DMARD naїve MTX treated patients. Secondly the X-ray examinations for calculating radiological progression were made not exactly at the 12th month of treatment which could have minimally distorted the value of radiological progression at 12 months assumed as linear in the analysis. Thirdly the relatively low number of early RA cases with available X-rays, especially in a subanalysis may have reduced the statistical power of our calculations. All of these limitations stem from the fact that our analysed patients were treated in routine clinical care and according to the local guidelines, DMARD treatment is mandatory before starting anti-TNFs. Additionally the use of hands and feet X-rays for following radiological disease progression is not regular in routine care, especially not at exactly a time interval of 12 month: the fact that the availability of X-rays at two timepoints was an inclusion criteria may have indeed introduced a selection bias into our study.

## Conclusions

Our results prove the superiority of anti-TNF + MTX therapy in dissociating inflammation and radiologic destruction. Anti-TNF + MTX treatment strongly inhibited radiological progression even in patients not reaching clinical remission or low disease activity. Not only therapeutic responders, but also non-responders of the combination group showed better radiologic results than patients on MTX monotherapy. Our data indicates that even if anti-TNF + MTX treatment is only partly successful in achieving remission or low disease activity, it can inhibit radiographic damage.

Anti-TNF + MTX treatment strongly inhibited radiological progression even in patients not reaching clinical remission or low disease activity. Further “real-life” investigations are needed in the future to evaluate the impact of anti-TNF + MTX treatment on clinical and radiological dissociation in routine clinical practice.

## Abbreviations

anti-TNF: Anti-tumour necrosis factor; MTX: Methotrexate; RA: Rheumatoid arthritis; DAS28: Disease activity score based on 28 joints; HAQ-DI: Health assessment questionnaire disability index; vdHS: Van der Heijde modified Sharp method; DMARD: Disease modifying antirheumatic drug; CDAI: Clinical disease activity index; anti-CCP: Anti-cyclic citrullinated peptid; RANKL: Receptor activator of nuclear factor kB ligand; OPG: Osteoprotegerin; RCT: Randomised controlled trial; ADA: Adalimumab; IL-6: Interleukin-6.

## Competing interest

The authors declare that they have no competing interests.

## Authors’ contributions

All authors contributed in the data collection and manuscript preparation. AM, JB and PJ performed the scoring of the hands and feet X-rays. PJ participated in the statistical analysis. All authors approved the final version of the manuscript.

## Pre-publication history

The pre-publication history for this paper can be accessed here:

http://www.biomedcentral.com/1471-2474/15/251/prepub

## References

[B1] Myasoedova E Davis JM Crowson CS Gabriel SE Epidemiology of rheumatoid arthritis: rheumatoid arthritis and mortality Curr Rheumatol Rep 2010 12 379 385 20645137 10.1007/s11926-010-0117-y

[B2] Gonzalez A Kremers HM Crowson CS Nicola PJ Davis JM Therneau TM Roger VL Gabriel SE The widening mortality gap between rheumatoid arthritis patients and the general population Arthritis Rheum 2007 56 3583 3587 17968923 10.1002/art.22979

[B3] Kuper HH van Leeuwen MA van Riel PL Prevoo ML Houtman PM Lolkema WF van Rijswijk MH Radiographic damage in large joints in early rheumatoid arthritis: relationship with radiographic damage in hands and feet, disease activity, and physical disability Br J Rheumatol 1997 36 855 860 9291854 10.1093/rheumatology/36.8.855

[B4] Curtis JR Singh JA Use of biologics in rheumatoid arthritis: current and emerging paradigms of care (review) Clin Ther 2011 33 697 707 10.1016/j.clinthera.2011.05.044 PMC3707489 21704234

[B5] Smolen JS Aletaha D Bijlsma JWJ Breedveld FC Boumpas D Burmester G Combe B Cutolo M de Wit M Dougados M Emery P Gibofsky A Gomez-Reino JJ Haraoui B Kalden J Keystone EC Kvien TK McInnes I Martin-Mola E Montecucco C Schoels M van der Heijde D T2T Expert Committee Treating rheumatoid arthritis to target: recommendations of an international task force Ann Rheum Dis 2010 69 631 637 20215140 10.1136/ard.2009.123919 PMC3015099

[B6] Smolen JS Landewé R Breedveld FC Dougados M Emery P Gaujoux-Viala C Gorter S Knevel R Nam J Schoels M Aletaha D Buch M Gossec L Huizinga T Bijlsma JW Burmester G Combe B Cutolo M Gabay C Gomez-Reino J Kouloumas M Kvien TK Martin-Mola E McInnes I Pavelka K van Riel P Scholte M Scott DL Sokka T Valesini G EULAR recommendations for the management of rheumatoid arthritis with synthetic and biological disease-modifying antirheumatic drugs Ann Rheum Dis 2010 69 964 975 20444750 10.1136/ard.2009.126532 PMC2935329

[B7] Cush JJ Early rheumatoid arthritis - Is there a window of opportunity? J Rheumatol 2007 34 1 7 17985417

[B8] Breedveld F The value of early intervention in RA - a window of opportunity Clin Rheumatol 2011 30 33 39 10.1007/s10067-010-1638-5 21350796

[B9] Lipsky PE van der Heijde DM St. Clair EW Furst DE Breedveld FC Kalden JR Smolen JS Weisman M Emery P Feldmann M Harriman GR Maini RN Anti-Tumor Necrosis Factor Trial in Rheumatoid Arthritis with Concomitant Therapy Study Group Infliximab and methotrexate in the treatment of rheumatoid arthritis N Engl J Med 2000 343 1594 1602 11096166 10.1056/NEJM200011303432202

[B10] Keystone EC Schiff MH Kremer JM Kafka S Lovy M DeVries T Burge DJ Once-weekly administration of 50 mg etanercept in patients with active rheumatoid arthritis: results of a multicenter, randomized, double-blind, placebo-controlled trial Arthritis Rheum 2004 50 353 363 14872476 10.1002/art.20019

[B11] Weinblatt ME Keystone EC Furst DE Moreland LW Weisman MH Birbara CA Teoh LA Fischkoff SA Chartash EK Adalimumab, a fully human anti-tumor necrosis factor alfa monoclonal antibody, for the treatment of rheumatoid arthritis in patients taking concomitant methotrexate: the ARMADA trial Arthritis Rheum 2003 48 35 45 12528101 10.1002/art.10697

[B12] Keystone EC Genovese MC Klareskog L Golimumab, a human antibody to tumour necrosis factor α given by monthly subcutaneous injections, in active rheumatoid arthritis despite methotrexate therapy Ann Rheum Dis 2009 68 789 796 19066176 10.1136/ard.2008.099010 PMC2674549

[B13] Keystone EC van der Heijde DM Mason D Jr Landewé R Vollenhoven RV Combe B Emery P Strand V Mease P Desai C Pavelka K Certolizumab pegol plus methotrexate is significantly more effective than placebo plus methotrexate in active rheumatoid arthritis Arthritis Rheum 2008 58 3319 3329 18975346 10.1002/art.23964

[B14] Emery P Keystone EC Tony HP Cantagrel A van Vollenhoven R Sanchez A Alecock E Lee J Kremer J IL-6 receptor inhibition with tocilizumab improves treatment outcomes in patients with rheumatoid arthritis refractory to anti-tumour necrosis factor biologicals: Results from a 24-week multicentre randomised placebo-controlled trial Ann Rheum Dis 2008 67 1516 1523 18625622 10.1136/ard.2008.092932 PMC3811149

[B15] Cohen SB Emery P Greenwald MW Dougados M Furie RA Genovese MC Keystone EC Loveless JE Burmester GR Cravets MW Hessey EW Shaw T Totoritis MC REFLEX Trial Group Rituximab for rheumatoid arthritis refractory to anti-tumor necrosis factor therapy: Results of a multicenter, randomized, double-blind, placebo-controlled, phase III trial evaluating primary efficacy and safety at twenty-four weeks Arthritis Rheum 2006 54 2793 806 16947627 10.1002/art.22025

[B16] Smolen JS Han C Bala M Maini RN Kalden JR van der Heijde D Breedveld FC Furst DE Lipsky PE ATTRACT Study Group Evidence of radiographic benefit of treatment with infliximab plus methotrexate in rheumatoid arthritis patients who had no clinical improvement: A detailed subanalysis of data from the anti-tumor necrosis factor trial in rheumatoid arthritis with concomitant therapy study Arthritis Rheum 2005 52 1020 1030 15818697 10.1002/art.20982

[B17] Landewé R van der Heijde D Klareskog L van Vollenhoven R Fatenejad S Disconnect between inflammation and joint destruction after treatment with etanercept plus methotrexate: results from the trial of etanercept and methotrexate with radiographic and patient outcomes Arthritis Rheum 2006 54 3119 3125 17009230 10.1002/art.22143

[B18] Emery P Genovese MC van Vollenhoven R Sharp JT Patra K Sasso EH Less radiographic progression with adalimumab plus methotrexate versus methotrexate monotherapy across the spectrum of clinical response in early rheumatoid arthritis J Rheumatol 2009 36 1429 1441 19369462 10.3899/jrheum.081018

[B19] Smolen JS Avila JCM Aletaha D Tocilizumab inhibits progression of joint damage in rheumatoid arthritis irrespective of its anti-inflammatory effects: Disassociation of the link between inflammation and destruction Ann Rheum Dis 2012 5 687 693 22121130 10.1136/annrheumdis-2011-200395 PMC3329225

[B20] Aletaha D Alasti F Smolen JS Rituximab dissociates the tight link between disease activity and joint damage in rheumatoid arthritis patients Ann Rheum Dis 2013 72 7 12 22915619 10.1136/annrheumdis-2012-201970

[B21] Arnett FC Edworthy SM Bloch DA The American Rheumatism Association 1987 revised criteria for the classification of rheumatoid arthritis Arthritis Rheum 1988 31 315 324 3358796 10.1002/art.1780310302

[B22] Visser K Goekopp-Ruiterman YP de Vries-Bouwstra JK Ronday HK Seys PE Kerstens PJ Huizinga TW Dijkmans BA Allaart CF A matrix risk model for the prediction of rapid radiological progression in patients with rheumatoid arthritis receiving different dynamic treatment strategies: post hoc analyses from the BeSt study Ann Rheum Dis 2010 69 1333 1337 20498212 10.1136/ard.2009.121160

[B23] Hetland ML Christensen IJ Tarp U Dreyer L Hansen A Hansen IT Kollerup G Linde L Lindegaard HM Poulsen UE Schlemmer A Jensen DV Jensen S Hostenkamp G Østergaard M All Departments of Rheumatology in Denmark Direct comparison of treatment responses, remission rates, and drug adherence in patients with rheumatoid arthritis treated with adalimumab, etanercept, or infliximab: results from eight years of surveillance of clinical practice in the nationwide Danish DANBIO registry Arthritis Rheum 2010 62 22 32 20039405 10.1002/art.27227

[B24] De Punder YM Fransen J Kievit W Houtman PM Visser H van de Laar MA van Riel PL The prevalence of clinical remission in RA patients treated with anti-TNF: results from the Dutch Rheumatoid Arthritis Monitoring (DREAM) registry Rheumatology 2012 51 1610 1617 22539487 10.1093/rheumatology/kes078

[B25] Aletaha D Smolen JS Ward MM Measuring function in rheumatoid arthritis: identifying reversible and irreversible components Arthritis Rheum 2006 54 2784 2792 16947781 10.1002/art.22052

[B26] Aletaha D Funovits J Smolen JS Physical disability in rheumatoid arthritis is associated with cartilage damage rather than bone destruction Ann Rheum Dis 2011 70 733 739 21321002 10.1136/ard.2010.138693

[B27] Markatseli TE Voulgari PV Alamanos Y Drosos AA Prognostic factors of radiological damage in rheumatoid arthritis: A 10-year retrospective study J Rheum 2011 38 44 52 20952476 10.3899/jrheum.100514

[B28] Geusens P The role of RANK ligand/osteoprotegerin in rheumatoid arthritis Ther Adv Musculoskelet Dis 2012 4 225 233 22859921 10.1177/1759720X12438080 PMC3403250

[B29] Smolen JS Aletaha D Grisar J Redlich K Steiner G Wagner O The need for prognosticators in rheumatoid arthritis. Biological and clinical markers: where are we now? Arthr Res Ther 2008 10 208 18557991 10.1186/ar2418 PMC2483438

[B30] Smolen JS van der Heijde DM Keystone EC van Vollenhoven RF Goldring MB Guérette B Cifaldi MA Chen N Liu S Landewé RB Association of joint space narrowing with impairment of physical function and work ability in patients with early rheumatoid arthritis: protection beyond disease control by adalimumab plus methotrexate Ann Rheum Dis 2012 0 1 7 10.1136/annrheumdis-2012-201620 PMC3686261 22915617

[B31] Boers M van Tuyl L van den Broek M Kostense PJ Allaart CF Meta-analysis suggests that intensive non-biological combination therapy with step-down prednisolone (COBRA strategy) may also ‘disconnect’ disease activity and damage in rheumatoid arthritis Ann Rheum Dis 2013 72 406 409 23155223 10.1136/annrheumdis-2012-202333

